# The association between shift work and the incidence of reflux esophagitis in Korea: a cohort study

**DOI:** 10.1038/s41598-023-29567-z

**Published:** 2023-02-13

**Authors:** Min-Woo Nam, Yesung Lee, Eunchan Mun, Woncheol Lee

**Affiliations:** Department of Occupational and Environmental Medicine, Kangbuk Samsung Hospital, Sungkyunkwan University School of Medicine, 29 Saemunan-ro, Jongno-gu, Seoul, 03181 South Korea

**Keywords:** Gastroenterology, Health occupations

## Abstract

Shift work has adverse health effects such as diabetes, cardiovascular disease, sleep disturbance, depression, and breast cancer. Gastro-esophageal reflux disease (GERD) results in lesions such as reflux esophagitis (RE) and Barrett’s esophagus. This study investigated the association between shift work and RE. A cohort study was conducted with 140,553 participants who were followed up at least once from 2012 to 2018. Type of working and shift types were collected using standardized questionnaires. Esophagogastroduodenoscopy (EGD) was performed by experienced endoscopists who were blinded to the aims of this study. According to the Los Angeles classification, RE was categorized based on the extent of esophageal mucosal breaks. During the 469,217.2 person-years of follow-up, 35,185 participants developed incident cases of RE. The multivariable adjusted hazard ratio (95% confidence intervals) for incident cases comparing shift work to fixed day work was 1.09 (1.04–1.13). This association was more strongly observed in the younger age group (18–39 years old) and the female group. In conclusion, shift work was significantly associated with the incidence of RE. Particularly, the results were more significant in the younger and female groups.

## Introduction

Shift work is an important occupational health issue in South Korea. According to the 2017 5th Korean Working Environment Survey, 9.7% of workers engaged in shift work^1^. Furthermore, findings in previous studies show the adverse health effects of shift work, including cardiovascular disease^2^, depression^3^, sleep disorder^4^, breast cancer^5^.

Gastro-esophageal reflux disease (GERD) is an abnormal reflux of gastric contents into the esophagus^6^. It results in reflux symptoms, including regurgitation and heartburn, and lesions such as reflux esophagitis (RE) and Barrett’s esophagus^6,7^. The prevalence of RE was 3.4% in 1997, increased to 6.2% in 2008, and continues to increase in South Korea^6^. In previous studies, risk factors for RE include age^8,9^, alcohol consumption^10^, smoking^9,11^ and obesity^9,11,12^.

Recently, a cross-sectional study was conducted on the association between shift work and RE at the Kangbuk Samsung Hospital^13^. We conducted a cohort study on the association between shift work and RE using endoscopic findings to confirm this association. This large-scale study was conducted in a relatively healthy adult population, using a cohort constructed from regular comprehensive health screening data.

## Methods

### Study population

The Kangbuk Samsung Cohort Study is a cohort study of population who underwent comprehensive health examination at the Kangbuk Samsung Hospital Total Health Care Center^14,15^. This retrospective cohort study was conducted from January 2002 to March 2012, and the Kangbuk Samsung Hospital officially started a prospective cohort study in April 2012^14,15^.

**Figure 1:**
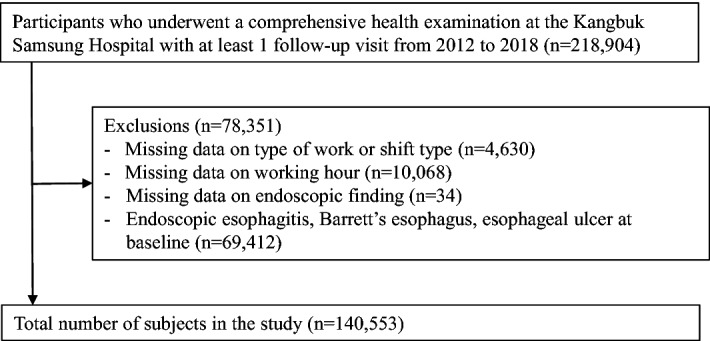
Flow chart of study participants.

The present study included participants who underwent a comprehensive health examination at Kangbuk Samsung Hospital with at least one follow-up visit from 2012 to 2018^15^ (n = 218,904). We excluded participants who had any of the following conditions at baseline^15^: missing data on the type of work or shift type (n = 4630), missing data on endoscopic finding (n = 34), endoscopic esophagitis, Barrett’s esophagus, or esophageal ulcer at baseline (n = 69,412), and missing data on working hours (n = 10,068). The total number of participants in the study^15^ was 140,553 (Fig. 1).

The Institutional Review Board of Kangbuk Samsung Hospital approved this study (approval number: KBSMC 2022-03-011) and waived the requirement for informed consent because we only used de-identified data obtained as part of routine health screening examinations^13,16^. All methods of the study were performed in accordance with the relevant guidelines and regulations including Declaration of Helsinki.

### Data collection

All examinations were conducted at Kangbuk Samsung Hospital Total Health Care Center clinics in Seoul and Suwon, South Korea^15^. Medical history, medication, working hours, type of work (fixed day work or shift work), shift type (fixed shift, rotating shift, others), alcohol consumption, smoking status, exercise frequency, and education level were collected at each clinical visit using standardized questionnaires. The question, “In the past year, what time of day have you mostly worked?” measured the type of work. The following responses were possible: “I usually work during the day (between 6 a.m. and 6 p.m.)^13^ or “I work in a different time slot.” We classified participants who answered with the latter as shift workers. The shift type includes fixed shift work (evening shift or night shift), rotating shift work (regular day and night shifts, 24 h shifts, and irregular shift work), and others (such as split shift)^13^.

Alcohol consumption was categorized into nondrinking, light drinking (< 10 g/day), or moderate-to-heavy drinking (> 10 g/day)^15^. Smoking status was categorized as never smoked, former smoker, or current smoker^15^. Regular exercise refers to exercising three or more times per week^16^. Education level was divided into non-collegiate education and a college graduate^16^.

Height and weight were measured by well-trained nurses, with participants wearing a lightweight hospital gown and no shoes^15^. Height was measured to the nearest 1 mm using a stadiometer, with the participant standing barefoot^15^. Weight was measured to the nearest 0.1 kg using a bioimpedance analyzer (InBody 3.0, Inbody 720, Biospace Co., Seoul, Korea)^15^. Body mass index (BMI) was calculated as the weight in kilograms divided by the height in meters squared^15^. BMI was categorized based on the criteria established for Asian populations: underweight, BMI < 18.5 kg/m^2^; normal-weight, BMI = 18.5–23 kg/m^2^; overweight, BMI = 23–25 kg/m^2^; obesity, BMI ≥ 25 kg/m^2^^17^. Blood specimens were collected from the antecubital vein after at least 10 h of fasting^15^. Diabetes was defined as a glycated hemoglobin concentration (HbA1c) ≥ 6.5%. Dyslipidemia was defined as the current use of antidyslipidemic drugs.

### Measurement and classification of reflux esophagitis

Experienced gastroenterology specialists performed esophagogastroduodenoscopy using the Evis Lucera CV-260 endoscope (Olympus Medical Systems, Tokyo, Japan)^18^. They evaluated the severity of RE using the Los Angeles (LA) classification^13,19^. Esophagitis is classified as LA-M (no mucosal injury, but redness with unclear boundaries), LA-A (mucosal break no longer than 5 mm, limited to the mucosal folds), LA-B (mucosal break more than 5 mm; mucosal injuries on different mucosal folds are not continuous with each other), LA-C (mucosal break continuous for two or more mucosal folds but which involves less than 75% of the circumference), LA-D (mucosal break which involves at least 75% of the esophageal circumference), and LA classification with Japanese modification including minimal change grade (LA-M)^13,19^.

### Statistical analyses

The study participants’ characteristics were explored according to their type of work. P values were calculated using t-test for continuous variables and chi-square test for discrete variables. We started follow-up with all participants free of RE, Barrett’s esophagus, and esophageal ulcer at baseline, and the endpoint was incident RE. Participant follow-up was extended from the baseline examination until the development of RE or the last health examination conducted before December 31, 2018, whichever came first^15^. Incidence rates were calculated as the number of incident cases divided by the person-years of follow-up^15^.

We estimated adjusted hazard ratios (HRs) with 95% confidence intervals (CIs) for incident cases of RE by comparing the type of work at baseline with fixed day work^15^. We used a continuous variable with the category number to determine the linear risk trends and tested its statistical significance in the regression models^15^. The models were initially adjusted for sex and age and then further adjusted for alcohol intake, smoking status, regular exercise, education level, BMI, diabetes, dyslipidemia, and working hours. We also conducted a subgroup analysis to identify^15^ the effects of sex and age. Statistical significance was set at *p* < 0.05 (two-tailed)^15^. We used STATA version 17.0 (Stata Corp., College Station, TX, USA) for data analysis^15^.

## Results

The baseline characteristics of the study participants are presented in Table 1. The mean (standard deviation) age and BMI of the 140,553 participants were 37.9 (7.5) years and 23.5 (3.3) kg/m^2^, respectively. Of all the participants, 66.4% were male, and 51.8% of shift workers were male. Furthermore, shift workers were more likely to be younger, drink alcohol and exercise, and be less likely to smoke and be obese. Finally, shift workers had a relatively low level of education, and the proportion of patients with diabetes and dyslipidemia was small.

**Table 1:** Baseline characteristics of study participants by reported type of work.

Characteristics	Overall	Type of work		*p* value
		Fixed day work	Shift work	
Number	140,553	128,910	11,643	
Age (years)^a^	37.9 (7.5)	38.3 (7.4)	33.7 (7.6)	< 0.001
Male worker (%)	66.4	67.7	51.8	< 0.001
Moderate-to-heavy alcohol drinking (%)^b^	16.6	16.5	17.9	< 0.001
Current smoker (%)	21.9	22.2	18.5	< 0.001
Regular exercise (%)^c^	12.4	12.1	15.4	< 0.001
Education (%) (≥ college graduate)	85.6	87.9	59.9	< 0.001
Obesity (%)	29.8	30.0	27.8	< 0.001
BMI (kg/m^2^)^a^	23.5 (3.3)	23.5 (3.2)	23.2 (3.6)	< 0.001
Diabetes mellitus (%)	3.0	3.1	1.8	< 0.001
Dyslipidemia (%)	2.6	2.7	1.3	< 0.001

*BMI* body mass index.

^a^Data are presented as the means (standard deviation).

^b^Data are presented as percentage (≥ 10 g/day).

^c^Data are presented as percentage (≥ 3 times per week).

We identified 35,185 incident cases of RE during 469,217.2 person-years of follow-up (incident rate, 75.0/1000 person-years). The average follow-up period for the participants was 3.3 years. Table 2 shows the association between shift work and the incidence of RE. The adjusted HR (95% CIs) for incident cases with RE comparing shift work to fixed-day work was 1.09 (1.05–1.13) in a sex-age-adjusted model. In a multivariate model adjusting for sex, age, smoking status, alcohol intake, regular exercise, education level, BMI, diabetes, dyslipidemia, and working hours, the adjusted HR (95% CIs) for incident cases with shift work compared to fixed day work was 1.07 (1.02–1.11). There was a significant association between shift work and incident cases with RE. The association between fixed shift work and RE in the multivariate model was not statistically significant. However, there was a significant association with rotating shift work. The adjusted HR (95% CIs) for incident cases with RE comparing rotating shift work to fixed day work was 1.08 (1.02–1.14) in a multivariate model.

**Table 2:** Development of reflux esophagitis (RE) by type of work and shift type in participants.

Type of work		Person-years	Number of incident cases	Incidence rate (per 1000 person-years)	Sex-age-adjusted HR	Multivariable-adjusted HR^a^ (95% CI)
Fixed day work		431,882.4	32,444	75.1	1.00 (reference)	1.00 (reference)
Shift work		37,334.8	2741	73.4	1.09 (1.05–1.13)	1.07 (1.02–1.11)
*p* for trend					< 0.001	0.003
Shift type	Fixed shift	4752.8	310	65.2	1.01 (0.91–1.13)	1.00 (0.89–1.13)
	Rotating shift	23,135.9	1730	74.8	1.10 (1.05–1.15)	1.08 (1.02–1.14)
	others	9446.2	701	74.2	1.11 (1.03–1.19)	1.07 (0.99–1.15)

*CI* confidence interval, *HR* hazard ratio.

^a^Adjusted for sex, age, smoking status, alcohol intake, regular exercise, education level, BMI, Diabetes, dyslipidemia and working hours.

The association between shift work and RE was more strongly observed in younger participants (18–39 years old). In younger participants, a multivariate model showed that the adjusted HR (95% CIs) for incident cases with RE comparing shift work to fixed day work was 1.13 (1.08–1.19). In contrast, in older participants (over 40 years old), there was no significant association between shift work and the incidence of RE (Table 3). This association was stronger in female workers. In female workers, the adjusted HR (95% CIs) for incident cases with RE comparing shift work to fixed day work was 1.13 (1.05–1.22) in a multivariate model. In male workers, the adjusted HR (95% CIs) for incidents with RE comparing shift work to fixed day work was 1.05 (1.00–1.11), so there was no statistically significant association (Table 3). We conducted an analysis on only the participants who maintained their work schedule during the follow-up. As a result, there was still a significant association between incidence of RE and shift work in the multivariable adjusted model. The adjusted HR for incident cases with RE comparing shift work to fixed-day work was 1.23 (1.17–1.31) in the multivariable adjusted model. We added this result to supplementary Table 1. Also, we analyzed the association except for LA-M. As a result, there was still a significant association between incidence of RE and shift work. The adjusted HR for incident cases with RE comparing shift work to fixed day work was 1.10 (1.00–1.21) in the multivariable adjusted model. We added this result to supplementary Table 2. Finally, we conducted an analysis excluding patients using digestive medicine. The adjusted HR for incident cases with RE comparing shift work to fixed day work was 1.06 (1.02–1.11) in the multivariable adjusted model. We added this result to supplementary Table 3.

**Table 3:** Subgroup analysis of development of reflux esophagitis (RE) by type of work according to sex and age.

Type of work	Person-years	Number of incident cases	Incidence rate (per 1000 person-years)	Multivariable-adjusted HR^a^ (95% CI)
18–39 years old				
Fixed day work	264,340.5	19,787	74.9	1.00 (reference)
Shift work	30,023.6	2209	73.6	1.13 (1.08–1.19)
* p* for trend				< 0.001
Over 40 years old				
Fixed day work	167,541.9	12,657	75.6	1.00 (reference)
Shift work	7311.2	532	72.8	1.02 (0.93–1.12)
* p* for trend				0.673
Female				
Fixed day work	133,769.7	7048	52.7	1.00 (reference)
Shift work	18,190.3	1058	58.2	1.13 (1.05–1.22)
* p* for trend				< 0.001
Male				
Fixed day work	298,112.7	25,396	85.2	1.00 (reference)
Shift work	19,144.5	1683	87.9	1.05 (1.00–1.11)
* p* for trend				0.070

*CI* confidence interval, *HR* hazard ratio.

^a^Adjusted for sex, age, smoking status, alcohol intake, regular exercise, education level, BMI, Diabetes, dyslipidemia and working hours.

## Discussion

We compared the incidence of RE in a shift work group and a fixed-day work group of healthy adult participants. We found a statistically significant association between shift work and the incidence of RE.

Several previous studies have investigated the association between shift work and gastro-intestinal symptoms and diseases. According to a study on male shipyard workers in Korea, night shift work is a risk factor for erosive esophagitis^20^. According to a study on electronics company workers, shift workers experienced endoscopic gastritis more than non-shift workers^21^. A Chinese study of 2027 workers found that rotating night shift work is independently associated with an increased risk of GERD symptoms^22^. According to Mun et al., there was a cross-sectional association between shift work and mild RE (≤ LA-A) compared with daily fixed time shifts^13^. However, most previous studies were cross-sectional, and few studies have observed endoscopic findings.

The circadian rhythm affects major gastrointestinal functions, such as gut motility, gastric acid secretion, and digestive enzyme production. Changes in circadian rhythm due to shift work are associated with gastrointestinal diseases such as GERD, peptic ulcer disease, and irritable bowel syndrome. Biological rhythms controlled by the hypothalamus's suprachiasmatic nucleus (SCN) play an important role in physiological, gastrointestinal, and liver functions^23^. Melatonin also plays an important role in the regulation of circadian rhythm. It re-synchronizes the SCN by providing information on light and darkness^23^. The eating habits of shift workers are also a factor that causes gastrointestinal symptoms. They have irregular meal-times because of changes in their work schedules. They sometimes skip meals and have a higher meal intake at night^24^. Such habits are likely to cause gastrointestinal problems.

According to a previous cross-sectional study at Kangbuk Samsung Health Study^13^, there was a significant association between RE and rotating shift work. In contrast, it was not found in fixed shift work. Similarly, in our study, the association between rotating shift work and the incidence of RE was significant; however, there was no significant association with fixed shift work. This result reinforces the conclusions of a previous cross-sectional study. This may be related to meal time. In the case of fixed night workers, meals can be eaten at a relatively constant time, but rotating shift workers have irregular meals due to their work schedules. Therefore, rotating shift workers seem to be more vulnerable to the risk of RE than fixed shift workers.

The prevalence of GERD increases with age^25^. This is related to age-related changes in the lower esophageal sphincter (LES). The LES is a short segment of the contracted smooth muscle at the distal end of the esophagus^26^. They play an essential role in swallowing^27^. GERD’s prevalence increases as the LES’s length and pressure decrease and esophageal motility is impaired with aging^25,28^. In addition, older individuals often have several factors that increase their risk for RE such as Helicobacter pylori infection, smoking, or use of medications (e.g., nonsteroidal anti-inflammatory drugs (NSAIDs))^29^. In our study, in individuals over 40 years of age, there was no significant association between shift work and the incidence of RE. However, in the case of 18–39 years of age, the incidence of RE was significantly higher in the shift workers. This shows that shift work is a strong risk factor for RE, excluding age-related deterioration of the LES. In addition, it seems that the younger age group, who is relatively less affected by factors such as Helicobacter pylori infection, smoking, or taking NSAIDs, is more affected by shift work to the incidence of RE than older participants.

Differences between men and women in the association between shift work and RE have not been clearly identified. In our study, the association between shift work and the incidence of RE was significant in women but not in men. The relationship between shift work and female hormones may have led to this result. Estrogen modulates fat metabolism, and obesity is a risk factor for RE^30^. Furthermore, Schwarz et al. reported that night shift work could affect female hormonal balance^31^. Exposure to light at night results in lower melatonin levels, which may be involved in regulating gonadal function by influencing the hypothalamic-pituitary–gonadal axis^31^. Considering these studies, it seems that women are more affected by RE associated with shift work than men.

We conducted an analysis excluding patients using digestive medicine. Digestive medicine includes digestives and antacids. As a result, still statistically significant results were obtained. We showed this result in supplementary Table 3.

Our study had several limitations. First, the participants could answer different types of work for each follow-up^15^. However, the participants maintained their work schedule during the follow-up in most cases. We conducted an analysis on only the participants who maintained their work schedule during the follow-up. As a result, there was still a significant association between incidence of RE and shift work in the multivariable adjusted model. We showed this result in supplementary Table 1. Second, as participants self-reported the questionnaire about the type of work, information bias could occur. Nevertheless, the misclassification is likely to be non-differential, because there is no gain in answering which type of work^15,32^. Third, there may be differences in judgement among gastroenterology endoscopists in the diagnosis and classification of RE. However, they evaluated RE as closely as possible to the Los Angeles classification. Finally, Helicobacter pylori infection was not included in the statistical analysis because Helicobacter pylori infection was not always tested, but only when *Helicobacter pylori* infection was suspected or the patients themselves wanted.

On the other hand, our study has several strengths. First, because our study was a cohort study with a large sample size, it had a high level of evidence for temporal causality^15^. In addition, most previous studies on the association between shift work and RE have been cross-sectional. Second, as our study was conducted on relatively healthy and young participants (mean age = 37.9 years), the effects of underlying diseases could be quite excluded^15^. Third, we investigated whether shift work had a more significant association with the incidence of RE in the younger and female groups through a subgroup analysis. Finally, since RE was analyzed as an endoscopic finding rather than as a symptom or questionnaire, more objective results could be derived.

In conclusion, shift work was significantly associated with the incidence of RE in our study. Particularly, the results were more significant in the younger and female groups.

## Supplementary Information

Supplementary Tables.

## Data Availability

The data are not available to be shared publicly because we do not have a permission from the IRB to distribute the data. However, analytical methods are available from corresponding author on reasonable request.
