# Supplementary material for: The association between shift work and the incidence of reflux esophagitis in Korea: a cohort study
Source: Sci Rep. 2023 Feb 13;13:2536. doi: 10.1038/s41598-023-29567-z (PMC9925718; doi:10.1038/s41598-023-29567-z)
Supplement: Supplementary file 1 — Supplementary Tables. [file 41598_2023_29567_MOESM1_ESM.docx]

**Supplementary table 1.** Development of reflux esophagitis (RE) by type of work in participants who maintain their work schedule during the follow-up. [CI = confidence interval; HR = hazard ratio]

| Type of work | Person-years | Number of incident cases | Incidence rate (per 1,000 person-years) | Sex-Age-adjusted HR | Multivariable-adjusted HR^a^ (95% CI) |
| --- | --- | --- | --- | --- | --- |
| Fixed day work | 416,548.4 | 31,575 | 75.8 | 1.00 (reference) | 1.00 (reference) |
| Shift work | 19,296.4 | 1,512 | 78.4 | 1.25 (1.19-1.32) | 1.23 (1.17-1.31) |
| *p* for trend |  |  |  | <0.001 | <0.001 |

^a^Adjusted for sex, age, smoking status, alcohol intake, regular exercise, education level, BMI, Diabetes, dyslipidemia and working hours

**Supplementary table 2.** Development of RE (except for LA-M) by type of work in participants. [CI = confidence interval; HR = hazard ratio]

| Type of work | Person-years | Number of incident cases | Incidence rate (per 1,000 person-years) | Sex-Age-adjusted HR | Multivariable-adjusted HR^a^ (95% CI) |
| --- | --- | --- | --- | --- | --- |
| Fixed day work | 431,882.4 | 7,205 | 16.7 | 1.00 (reference) | 1.00 (reference) |
| Shift work | 37,334.8 | 542 | 14.5 | 1.12 (1.02-1.22) | 1.10 (1.00-1.21) |
| *p* for trend |  |  |  | 0.013 | 0.041 |

^a^Adjusted for sex, age, smoking status, alcohol intake, regular exercise, education level, BMI, Diabetes, dyslipidemia and working hours

**Supplementary table 3.** Development of reflux esophagitis (RE) by type of work in participants except for patients using digestive medicine. [CI = confidence interval; HR = hazard ratio]

| Type of work | Person-years | Number of incident cases | Incidence rate (per 1,000 person-years) | Sex-Age-adjusted HR | Multivariable-adjusted HR^a^ (95% CI) |
| --- | --- | --- | --- | --- | --- |
| Fixed day work | 422,181.0 | 31,709 | 75.1 | 1.00 (reference) | 1.00 (reference) |
| Shift work | 36,610.0 | 2,683 | 73.3 | 1.09 (1.04-1.13) | 1.06 (1.02-1.11) |
| *p* for trend |  |  |  | <0.001 | 0.007 |

^a^Adjusted for sex, age, smoking status, alcohol intake, regular exercise, education level, BMI, Diabetes, dyslipidemia and working hours
